# Phthalate Acid Esters (PAEs) in Indoor Dust from Decoration Material Stores: Occurrence, Sources, and Health Risks

**DOI:** 10.3390/toxics12070505

**Published:** 2024-07-13

**Authors:** Li-Bo Chen, Chong-Jing Gao, Ying Zhang, Hao-Yang Shen, Xin-Yu Lu, Cenyan Huang, Xiaorong Dai, Jien Ye, Xiaoyu Jia, Kun Wu, Guojing Yang, Hang Xiao, Wan-Li Ma

**Affiliations:** 1 College of Biological & Environmental Science, Zhejiang Wanli University, Ningbo 315100, China; lbchen0107@163.com (L.-B.C.); z_yingzy@163.com (Y.Z.); shenhaoy2023@163.com (H.-Y.S.); xy_lu2021@163.com (X.-Y.L.); cyhuang@zwu.edu.cn (C.H.); xrdai@zwu.edu.cn (X.D.); yejien93@163.com (J.Y.); guojing_yang@163.com (G.Y.); 2 State Key Laboratory of Urban Water Resource and Environment, Harbin Institute of Technology, Harbin 150090, China; 3 Institute of Urban Environment, Chinese Academy of Sciences, Ningbo Observation and Research Station, Ningbo 315830, China; xyjia@iue.ac.cn (X.J.); kwu@iue.ac.cn (K.W.); 4 Institute of Urban Environment, Center for Excellence in Regional Atmospheric Environment, Chinese Academy of Sciences, Xiamen 361021, China; hxiao@iue.ac.cn; 5 Ningbo (Beilun) Zhongke Haixi Industrial Technology Innovation Center, Ningbo 315021, China; 6 State Key Laboratory of Urban Water Resource and Environment, Harbin Institute of Technology, International Joint Research Center for Persistent Toxic Substances (IJRC-PTS), Harbin 150090, China; 7 Heilongjiang Provincial Key Laboratory of Polar Environment and Ecosystem (HPKL-PEE), Harbin 150090, China

**Keywords:** phthalates, decoration materials, dust, indoor environment, health risk

## Abstract

Phthalate acid esters (PAEs) are one of the most widely used plasticizers globally, extensively employed in various decoration materials. However, studies on the impact of these materials on indoor environmental PAE pollution and their effects on human health are limited. In this study, forty dust samples were collected from four types of stores specializing in decoration materials (flooring, furniture boards, wall coverings, and household articles). The levels, sources, exposure doses, and potential health risks of PAEs in dust from decoration material stores were assessed. The total concentrations of Σ_9_PAE (the sum of nine PAEs) in dust from all decoration-material stores ranged from 46,100 ng/g to 695,000 ng/g, with a median concentration of 146,000 ng/g. DMP, DEP, DBP, and DEHP were identified as the predominant components. Among all stores, furniture board stores exhibited the highest Σ_9_PAE (159,000 ng/g, median value), while flooring stores exhibited the lowest (95,300 ng/g). Principal component analysis (PCA) showed that decoration materials are important sources of PAEs in the indoor environment. The estimated daily intakes of PAEs through non-dietary dust ingestion and dermal-absorption pathways among staff in various decoration-material stores were 60.0 and 0.470 ng/kg-bw/day (flooring stores), 113 and 0.780 ng/kg-bw/day (furniture board stores), 102 and 0.510 ng/kg-bw/day (wall covering stores), and 114 and 0.710 ng/kg-bw/day (household article stores). Particularly, staff in wall-covering and furniture-board stores exhibited relatively higher exposure doses of DEHP. Risk assessment indicated that although certain PAEs posed potential health risks, the exposure levels for staff in decoration material stores were within acceptable limits. However, staff in wall covering stores exhibited relatively higher risks, necessitating targeted risk-management strategies. This study provides new insights into understanding the risk associated with PAEs in indoor environments.

## 1. Introduction

The interior environment plays a pivotal role in people’s daily lives, serving as a crucial space for living and working. With most people spending more than 90% of their time indoors, the quality of the indoor environment significantly impacts human health [1,2]. Decoration material is an important factor that affects indoor environmental quality. In the pursuit of enhanced comfort and aesthetics, people increasingly devote attention to the decoration of interior space nowadays, involving the utilization of diverse decoration materials such as flooring, furniture board, paint, and wall covering to embellish indoor spaces. However, the extensive use of these decoration materials raises concerns about the potential escalation of indoor pollution and its consequential threat to human health. A large number of studies underscore the fact that decoration materials are permanent release sources of various pollutants (formaldehyde, benzene, radionuclides, e.g., [3,4,5]. Human exposure to these chemicals can result in respiratory problems, chronic cough, asthma and recurrent respiratory infection, and even a carcinogenic effect [6,7]. Given the alarming implications of pollutants present in decoration materials on both the indoor environment and human health, substantial attention should be devoted to this issue.

Phthalate acid esters (PAEs) are a class of synthetic environmental disrupting chemicals (EDCs). As the most extensively utilized additives, PAEs are widely used in a variety of decoration materials, which impart favorable characteristics such as flexibility, transparency, durability, and longevity [8]. Usually, long alkyl chain PAEs, including bis(2-ethylhexyl) phthalate (DEHP), di-iso-nonyl phthalate (DINP) and bis(2-propylheptyl) phthalate (DPHP), are incorporated into polyvinyl chloride (PVC) decoration products such as flooring, furniture boards and PVC pipes to enhance material flexibility [9]. Conversely, those with shorter chains, such as di-n-butyl phthalate (DBP), di-iso-butyl phthalate (DIBP), butyl-benzyl phthalate (BBP), diethyl phthalate (DEP) and dimethyl phthalate (DMP), find predominant usage in non-PVC decoration products like paints, textiles, lubricants, and adhesives to augment material lubricity [10,11]. PAEs added to decoration materials do not covalent bond with the matrix materials, but are instead adhered to decoration materials through Johannes Diderik Van der Waals forces. Consequently, they have the potential to migrate from the materials into the surrounding environment, leading to environmental pollution and adverse effects on human health [12,13,14]. Epidemiological studies have consistently revealed a significant correlation between human exposure to PAEs and various health tissues related to the reproductive system diseases, type II diabetes, insulin resistance, overweight/obesity, allergies and asthma [15]. Functioning as EDCs, PAEs (especially DBP and DEHP) also contribute to a decline in sperm quality, a decrease in testosterone levels, and damage to sperm DNA [16,17]. Furthermore, PAEs can modify ovarian and oocyte development, disrupt follicular development [18], and advance puberty in girls [19].

PAEs are significant pollutants in indoor environments. The contamination by PAEs in various indoor settings has been extensively documented [20]. Nevertheless, these studies predominantly focused on households [21], student dormitories [22], offices [23], and kindergartens [24,25]. Occupational exposure settings, especially those related to decorative materials, remain notably underrepresented [26,27]. In this study, PAEs in indoor dust sourced from four types of decoration material stores were analyzed. The objectives of this study are to (1) analyze the characteristics of PAE contamination in indoor dust of decoration material stores; (2) identify the sources of PAEs in indoor dust originating from different decoration materials; and (3) elucidate the impact of decorative materials on PAE exposure and its health risk for the working population.

## 2. Materials and Methods

### 2.1. Reagents and Chemicals

Nine target PAEs including dimethyl phthalate (DMP), diethyl phthalate (DEP), di-n-butyl phthalate (DBP), di-iso-butyl phthalate (DIBP), butyl-benzyl phthalate (BBP), dicyclohexyl phthalate (DCHP), di-hexyl phthalate (DHxP), bis(2-ethylhexyl) phthalate (DEHP), and di-n-octyl phthalate (DOP) and their isotope internal standards (D_4_-DMP, D_4_-DEP, D_4_-DBP, D_4_-DIBP, D_4_-BBP, D_4_-DCHP, D_4_-DHxP, D_4_-DEHP, and D_4_-DOP) were purchased from Accustandard (New Haven, CT, USA), Dr. Ehrenstorfer GmbH (Augsburg, Germany) and Anpel Laboratory Technologies (Shanghai) Inc. (Shanghai, China). The purity of all compounds is ≥ 98%. The solvents for extraction and instrument analysis, including methanol, n-hexane and ultrapure water, were purchased from Thermo Fisher Technologies Co., Ltd. (Shanghai, China).

### 2.2. Sample Collection, Preparation and Instrumental Analysis

From January 2019 to December 2020, a total of 40 indoor dust samples were collected from four types of decoration material stores including flooring stores (*n* = 8), furniture board stores (*n* = 14), wall covering stores (*n* = 7) and household article stores (*n* = 11). In selecting decoration material stores for our study, we aimed to ensure representation across several criteria. Firstly, we considered the geographical distribution, to encompass diverse urban and suburban areas. Secondly, we targeted a variety of stores that offer specific types of indoor-decoration material such as flooring, furniture board, wall covering and paint and coatings. Our selection criteria also emphasized the inclusion of stores that are recognized for their influence in the market and were willing to participate in the study. Sample sizes were determined based on availability and cooperation from the stores. For our sampling strategy, flooring, furniture board, and wall covering samples were obtained from display samples available in the stores. Paint and coating samples, on the other hand, were purchased directly from the stores to ensure they were fresh and representative of current stock. This approach was designed to capture a representative cross-section of indoor-decoration material sources, reflecting the diversity and typicality within our study area. Simultaneously, 10 dust samples were collected from ordinary households as controls. They were collected from 10 residences located in residential areas adjacent to indoor-decoration material stores. This selection criterion aimed to minimize potential outdoor environmental influences on indoor PAE levels by ensuring proximity to the sources of interest. Specific criteria included selecting residences where dust samples were predominantly collected from living rooms without wooden flooring and from houses that were not newly decorated but had undergone decoration more than 10 years prior to this. This approach ensured that our control samples were representative of typical residential environments adjacent to indoor-decoration material stores, enhancing the relevance and comparability of our findings within the scope of our study. Additionally, occupancy status criteria ensured that all selected households were currently inhabited. These measures were implemented to enhance the representativeness of the control group and facilitate meaningful comparisons with dust samples obtained from indoor-decoration material stores. The dust samples were collected using a portable vacuum cleaner. Prior to vacuuming, the pipe of the vacuum cleaner was purified by solvent to prevent cross-contamination between different dust samples during the collection process. Each dust sample was collected in an amount of at least 0.5 g. All dust samples were sealed in aluminum foil and stored at −20 °C in darkness. Sample pre-treatment was completed within one week of collection.

About 0.3 g of dust sample was transferred to a 12 mL glass tube and spiked with 250 ng of mixed internal standards. After equilibration for 2 h, the samples were added to 2.0 mL Milli-Q water and 4.0 mL hexane and then ultrasonically extracted for 30 min. The sample was further extracted by vigorously shaking for 1 h, followed by centrifuging at 4000 rpm for 10 min. The organic fraction was transferred into another cleaned glass tube. The sample was consequently added to 2.0 mL methanol and 4.0 mL hexane and extracted by vigorously shaking for 1 h followed by centrifuging at 4000 rpm for 10 min. The organic fraction was transferred. The combined organic fractions were concentrated to 0.5 mL under a gentle stream of nitrogen and stored at 4 °C for instrumental analysis. Nine target PAEs in dust were determined by using an Agilent Technologies 6890 gas chromatography system coupled with an Agilent Technologies 5975 mass spectrometer system. A fused-silica capillary column (DB-5; 30 m × 0.25 mm i.d.; 0.25 μm film thickness) was used to achieve chromatographic separation. The selected ion monitoring (SIM) mode was used. Detailed information on the instrumental analysis has been described in our previous study [28].

### 2.3. Quality Assurance and Quality Control (QA/QC)

For each batch of 20 samples, two method blanks, two spiked blanks and two pairs of matrix spike samples were processed. The recoveries of isotope internal standards in samples were 49 ± 29%, 65 ± 28%, 93 ± 29%, 100 ± 28%, 54 ± 22%, 56 ± 25%, 67 ± 30%, 79 ± 29% and 93 ± 47%. The recoveries of target compounds in matrix spike samples were 62–95%, respectively. Trace concentrations of DMP (2.07 ng/g), DEP (0.59 ng/g), DIBP (4.46 ng/g), DBP (5.74 ng/g) and DEHP (3.86 ng/g) were detected in the procedural blanks. The concentrations measured in the procedural blanks were subtracted from the concentrations. The limit of quantification (LOQ) for PAEs in dust samples is 2–10 ng/mL. Concentrations below the LOQ were assigned a value of LOD/√2 for statistical analysis.

### 2.4. Estimation of Human Exposure to PAEs

The measured concentrations of PAEs in indoor dust of decoration material stores were used to estimate the daily intakes (EDIs) of PAEs via non-dietary dust ingestion and dermal absorption from dust to skin. The equation for human exposure to PAEs via non-dietary dust ingestion is as follows:

(1) $${EDI}_{\mathrm{Ingestion}}=C_{\mathrm{dust}}\times f_{1}\times f_{2}/BW$$

where *EDI*_Ingestion_ is estimated daily intake via non-dietary dust ingestion (ng/kg-bw/day), *C*_dust_ is the concentration of PAEs in dust (ng/g), *f*_1_ is the indoor exposure fraction, *f*_2_ is the ingestion rate of indoor dust (g/day), and *BW* is the body weight (kg). The values of *f*_1_, *f*_2_ and *BW* used were 0.88, 0.05 g/day and 63 kg, as reported in the earlier study [22,29].

The equation for human exposure to PAEs via dermal absorption from dust to skin is

(2) $${\mathrm{EDI}}_{\mathrm{Dermal}\;\mathrm{absorption}}=C_{\mathrm{dust}}\times A\times M\times f_{1}\times f_{3}/\mathrm{BW}$$

where *EDI*_Dermal absorption_ is the estimated daily intake via dermal absorption from dust (ng/kg-bw/day), *C*_dust_ is the concentration of PAEs (ng/g), *A* is the surface area of exposure (cm^2^), *M* is the weight of media adhesion in contact with skin (mg/cm^2^/day), *f*_3_ is the dermal absorption rate of indoor dust, and *BW* is the body weight (kg). *A* is 4615 cm^2^ [29,30,31], *M* is 0.096 mg/cm^2^, and the values of *f*_4_ for different PAEs are 0.0004775 (DMP), 0.0010255(DEP), 0.000601 (DIBP), 0.000778 (DBP), 0.0003535 (BBP), and 0.000053 (DEHP) [29].

### 2.5. Carcinogenic Risk Assessment of DEHP

Carcinogenic risk (CR) of DEHP was estimated using the following equation, which was adapted from U.S. EPA [32]:

(3) $$\mathrm{CR}={\mathrm{EDI}}_{\mathrm{Ingestion}}\times \mathrm{SF}$$

where *CR* is the probability of developing cancer over a lifetime as a result of exposure to a contaminant, *EDI*_Ingestion_ is estimated daily intake of DEHP via dust ingestion, and *SF* is the slope factor for oral exposure (8.4 × 10^−3^ (mg/kg-bw/day)^−1^) [33].

### 2.6. Cumulative Risk Assessment of Exposure to Phthalates

Cumulative risk assessment for DIBP, DBP, and DEHP was estimated using the hazard index (HI), which is the sum of the hazard quotient (HQ) of individual PAEs, calculated with the following equations:

(4) $$\mathrm{HQ}=\frac{{\mathrm{EDI}}_{\mathrm{Ingestion}}}{\mathrm{Reference}\mathrm{limit}\;\mathrm{value}}$$

(5) $$\mathrm{HI}={\mathrm{HQ}}_{\mathrm{DIBP}}+{\mathrm{HQ}}_{\mathrm{DBP}}+{\mathrm{HQ}}_{\mathrm{DEHP}}$$

where *EDI*_Ingestion_ is the estimated daily intake via ingestion of each PAE, and the reference limit value is the reference dose (RfD) recommended by the United States Environmental Protection Agency (U.S. EPA) [34,35], or the reference dose for anti-androgenicity (RfD AA) developed by Kortenkamp and Faust [36]. The RfDs for DBP and DEHP were 100 μg/kg-bw/day and 20 μg/kg-bw/day, respectively [34,35]. Since U.S. EPA did not recommend an RfD for DIBP, it was adopted from Benson’s study with a value of 800 μg/kg-bw/day [37]. The RfD AA values for DIBP, DBP, and DEHP were 200 μg/kg-bw/day, 100 μg/kg-bw/day, and 30 μg/kg-bw/day, respectively [36]. It should be noted that both RfD and RfD AA values are estimated for oral exposure [34,35,36].

### 2.7. Risk Assessment of DEHP

No Significant Risk Level (NSRL) and Maximum Allowable Dose Levels (MADsL) were recommended by the Office of Environmental Health Hazard Assessment (OEHHA) of the United States [38]. NSRL is defined as “the daily intake level posing a 10^−5^ risk of cancer assuming lifetime exposure”, and MADL is defined as “the highest level at which the chemical would have no observable reproductive effect assuming exposure at 1000 times that level” [38]. Based on the optimization of NSRL and MADLs for DEHP recommended by OEHHA, China-specific NSRL and MADL for DEHP were developed to estimate the risk assessment of DEHP. The detailed calculation approach was referenced from an earlier study [39]:

(6) $$\mathrm{NSRL}=\frac{\mathrm{Cancer}\;\mathrm{Risk}\times \mathrm{BW}}{\mathrm{Cancer}\;\mathrm{Potency}\;\mathrm{Estimate}}$$

(7) $${\mathrm{NSRL}}_{\mathrm{adjusted},\;\mathrm{China}\;\mathrm{specific}}=\frac{{\mathrm{BW}}_{\mathrm{Chinese}}}{{\mathrm{BW}}_{\mathrm{American}}}\times \mathrm{NSRL}$$

where *BW*_Chinese_ is estimated as 63 kg and *BW*_American_ is estimated as 70 kg when calculating NSRL [40]; NSRL for DEHP is 310 μg/day [40].

The equation of MADL is the following [39,41]:

(8) $$\mathrm{MADL}=\frac{\mathrm{NOEL}\times \mathrm{BW}}{1000}$$

where the NOEL (No Observable Effect Level) for DEHP is 5800 μg/kg-bw/day [41], the *BW* value is estimated as 63 kg, and 1000 is the safety factor. It should be pointed out that NSRL for DEHP is estimated for both males and females via inhalation, non-dietary ingestion, and dermal absorption [40], whereas MADL for DEHP is estimated only for males via oral exposure [41].

Based on Equations (7) and (8), the risk quotients (RQs) of DEHP were estimated using the following equations:

RQ of DEHP based on China-specific NSRL is

(9) $${\mathrm{RQ}}_{\mathrm{NSRL}}=\frac{C\times f_{1}\times (f_{2}+A\times M\times f_{3})}{\frac{{\mathrm{BW}}_{\mathrm{Chinese}}}{{\mathrm{BW}}_{\mathrm{American}}}\times \mathrm{NSRL}}$$

RQ of DEHP based on China-specific MADL is

(10) $${\mathrm{RQ}}_{\mathrm{MADL}}=\frac{C\times {\;f}_{1}\times f_{2}}{\frac{\mathrm{NOEL}}{1000}\times {\mathrm{BW}}_{\mathrm{Chinese}}}$$

### 2.8. Data Analysis

SPSS Software (Version 22) was applied to perform the statistical analysis. Non-parametric tests (Kruskal–Wallis H Test and Mann–Whitney U Test) were used to compare concentration differences of all target compounds between different groups. Spearman’s rank correlation coefficients were used to analyze the relationship between two sets of data due to the limited sample size and the non-normal distribution of the data. Statistical significance was set at *p* < 0.05 with a two-tailed test.

## 3. Results and Discussion

### 3.1. Total Concentrations of PAEs in Decoration Material-Store Dust

All dust samples collected from decoration material stores contained detectable PAEs, indicating widespread presence of PAEs in decoration material stores. DMP, DEP, DBP, DIBP, and DEHP were consistently present compounds, with detection frequencies ranging from 82% to 100%. This is similar to those in household dust (80–100%). DCHP was detected in 63%, 50%, 57% and 45% of dust from flooring stores, furniture board stores, wall-covering and household-article stores, but it was not detected in household dust, indicating that decoration materials are closely related to DCHP pollution in indoor dust. In contrast to DCHP, DOP was seldom found in both decoration material stores and household dust. Among all decoration material stores, detectable dust DOP was only found in flooring stores (25%) and furniture board stores (7%).

The total concentrations of Σ_9_PAE (sum of nine PAEs) in the dust from all decoration material stores ranged from 46,100 ng/g to 695,000 ng/g, with a median concentration of 146,000 ng/g (Table 1). DEHP, DBP and DIBP were the predominant PAEs. They accounted in total for over 97% of Σ_9_PAE in all decoration material-store dust. The concentrations of DMP and DEP were relatively low. They were one or two orders of magnitude lower than those of DBP, DIBP and DEHP, and contributed to less than 3% of Σ_9_PAE. This contribution pattern is consistent with commonly reported findings that DBP, DIBP and DEHP are the most significant PAEs present in the dust of various indoor spaces (residential houses, offices, schools and other public places) [42,43,44]. The total concentrations of Σ_9_PAE in household dust ranged from 427,000 ng/g to 7,820,000 ng/g, with a median concentration of 1,220,000 ng/g. Compared to decoration material stores, household dust exhibited significantly higher Σ_9_PAE as well as a boarder concentration range. This is mainly because the household settings are more complex, and contains more PAE sources than decoration material stores.

### 3.2. Variations in PAEs in Different Decoration-Material Stores

**Figure 1:**
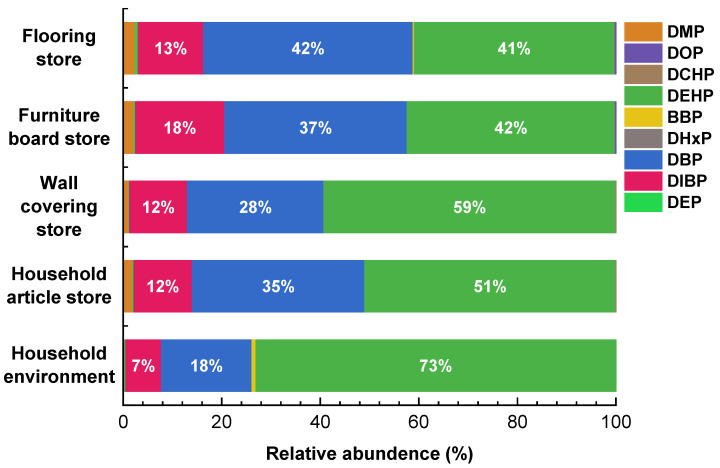
Characteristics of concentration composition of PAEs in the dust from different decoration-material stores and from the household environment.

The concentrations of PAEs in the dust from different decoration-material stores are summarized in Table 1 and Figure S1. The stores are ranked in ascending order of Σ_9_PAE, as follows: furniture board stores (159,000 ng/g, median value) > wall covering stores (146,000 ng/g) > household article stores (138,000 ng/g) > flooring stores (95,300 ng/g). Furniture board stores exhibited the highest Σ_9_PAE, while flooring stores exhibited the lowest. DEHP was the predominant PAE in all decoration material stores. The median concentrations of DEHP in flooring, furniture-board, wall-covering and household-article store dust were 56,000, 68,500, 94,900 and 62,200 ng/g, respectively. These concentrations were consistent across different stores and accounted for 41–59% of Σ_9_PAE (Figure 1). The concentration of DEHP in dust from household environments was 1,130,000 ng/g, which was significantly higher than that in decoration material stores (*p* < 0.05). DEHP in household dust also comprised a substantial proportion of the total Σ_9_PAE, accounting for approximately 73%.

DEHP, as a predominant PAE in dust, has been widely reported [45]. The elevated proportion of DEHP in dust from decoration material stores and household environments can be attributed, on the one hand, to its higher production and usage relative to PAEs in China [46], and on the other hand, to its high molecular weight, which facilitates its settling into dust [47,48]. Significantly higher levels of DEHP were found in household environments, suggesting the existence of DEHP sources in residential settings that are distinct from those found in decoration material stores. Previous studies have showed that flooring can lead to increased accumulation of DEHP in indoor floor dust [47,49]. However, this phenomenon was not found in the flooring store. The concentrations of DEHP in flooring stores were not observed to be significantly higher than those in the other three stores (*p* > 0.05). This is probably attributed to the fact that the dust samples collected in this study were exclusively marble-floor dust, whereas the floors displayed in flooring stores were mounted on walls and therefore had no significant impact on DEHP levels in ground dust.

The concentrations of DBP and DIBP in dust from flooring, furniture board, wall-covering and household-article stores were 37,700 and 12,900 ng/g, 45,600 and 22,500 ng/g, 27,400 and 21,500 ng/g, and 36,400 and 7030 ng/g, respectively. They together accounted for 55%, 55%, 40%, and 47% of the Σ_9_PAE. Significant concentration differences in DIBP were observed among different decoration stores (*p* > 0.05). Wall-covering and furniture-board stores exhibited significantly higher levels of DIBP (21,500 ng/g and 22,500 ng/g) compared to flooring stores (12,900 ng/g) and household article stores (7030 ng/g), suggesting the notable role of wall covering and furniture board in the accumulation of DIBP in the dust. The concentrations of DBP and DIBP in household dust were 72,100 ng/g and 27,300 ng/g. Significant differences in concentrations of DBP in the dust between the household environment and flooring stores (*p* < 0.05), wall covering stores (*p* < 0.05), and household article stores (*p* < 0.05) were observed; otherwise, it was observed that there were significant differences between concentrations of DIBP in the dust in the household environment and flooring stores (*p* < 0.05) and household article stores (*p* < 0.05).

DMP and DEP are widely detected in decoration material-store dust, but their concentrations were much lower compared to DEHP, DIBP and DBP. The concentrations of DMP in decoration material-store dust were significantly higher than that in household dust (979 ng/g). Additionally, significantly higher concentrations of DMP and DEP (3060 ng/g and 616 ng/g) were also found in flooring stores, surpassing those in wall covering stores (1160 ng/g and 199 ng/g). DMP and DEP are low-molecular-weight compounds primarily found in air rather than dust [48]. It has been reported that indoor air concentrations of DMP in China are relatively high, compared to other countries [50,51]. The exposure levels to DMP for Chinese residents are also significantly higher than those in other countries. However, the main source of DMP has not been identified [51]. Based on these results, we speculate that DMP in indoor environment in China may be closely related to decoration materials such as flooring, furniture board, and wall covering, suggesting that DMP could serve as an indicator reflecting PAE pollution originating from decoration materials.

The concentrations of DEP in dust from flooring, furniture board, wall covering and household article stores were 616 ng/g, 356 ng/g, 199 ng/g and 396 ng/g, respectively. They were significantly lower than that in household-environment dust (2090 ng/g) (*p* < 0.05). Previous studies have indicated that DEP mainly exists in personal care products [29,52,53,54,55]. Therefore, the relatively lower DEP levels in decoration stores may be attributed to the fact that personal care products are less commonly used in decoration material stores than in the household environment [30].

### 3.3. Comparison with Other Studies

Numerous studies have documented PAEs in indoor environments globally. Typically, DEHP, DBP and DIBP are the predominant PAEs found in indoor dust worldwide. Our findings in decoration material stores indicated relatively low DEHP levels compared to other studies. For instance, DEHP concentrations in household dust from other regions in China varied widely, ranging from 98.2 μg/g in Guangzhou (South China) to 1543 μg/g in Chongqing (Southwest China) [21,29,44,51,56,57,58,59,60,61,62,63,64]. Furthermore, the concentrations of DEHP in decoration material-store dust were also lower than those reported in dust from public places like classrooms (186 μg/g) [43], kindergartens (571.8 μg/g) [59], dormitories (134.9 μg/g) [62], commercial offices (1279 μg/g) [56], hospitals (707 μg/g) [57], shopping malls (958 μg/g) [57] and public micro-environments (684 μg/g) [44], as well as manufacturing plants (918 μg/g) [57], electronic factories (597 μg/g) [57] and e-waste recycling workshops (390 μg/g) [58]. Globally, DEHP concentrations in dust from decoration material stores were comparable to levels reported in Belgium (62 μg/g) [65], but significantly lower than those observed in California (172.2 μg/g) [39], Texas (155 μg/g) [66], the Northern United States (118.6 μg/g) [67], Canada (462 μg/g, 347 μg/g, 292 μg/g) [68], Sweden (680 μg/g, 449 μg/g, 130 μg/g) [69,70,71], the Netherlands (111 μg/g), Ireland (114 μg/g) [46], Germany (888 μg/g) [72], Turkey (316 μg/g) [73], South Korea (938 μg/g) [74], Thailand (3009 μg/g, 1479 μg/g, 1207 μg/g, 1739 μg/g) [75], Saudi (671.8 μg/g, 573.1 μg/g, 1020 μg/g, 790 μg/g) [46,76] and Kuwait (240 μg/g, 2256 μg/g) [76,77].

The concentrations of DIBP and DBP in our study were at medium levels. In furniture-board and wall-covering stores, they were higher than those in household dust from Shanghai (DIBP and DBP, 11.1 and 11.6 μg/g) [29], Guangzhou (10.4 and 9.3 μg/g) [29], and Taiwan (1.7 and 4.9 μg/g) [59], and comparable to values in Urumqi (32.8 and 170 μg/g) [29], Jinan (33.6 and 26.9 μg/g) [29] and Qingyuan (29 and 38 μg/g) [58]. Elevated levels of DIBP and DBP were notably found in Beijing (72.8 and 97 μg/g) [43], Nanjing (51.5 and 152.7 μg/g) [62], Chongqing (75.4 and 139.3 μg/g) [21] and Xi’an (233.8 and 134.77 μg/g) [42]. Internationally, DIBP and DBP levels in decoration-material dust were comparable to those in most countries but lower than those found in nursery and primary schools in France (52.6 and 38.2 μg/g) [78] and workplaces in Sweden (37 and 100 μg/g) [69]. The highest DIBP and DBP levels were found in Stockholm, with concentrations of DBP in homes (130 μg/g) [69], schools (150 μg/g) [69] and workplaces (100 μg/g) [69] significantly higher than those reported in other regions.

The concentrations of DMP in decoration material-store dust were notably elevated. They were significantly higher than those found in most household dust in China, as well as in dormitories (0.5 and 0.15 μg/g) [44,62], hospitals (0.43 μg/g) [57], manufacturing plants (0.24 μg/g) [57] and e-waste recycling workshops (0.23 μg/g) [58]. Additionally, DMP levels exceeded those reported for household and public environmental dust in most Asian countries, as well as in European and American countries, with the exception of nursery schools in Korea (2.1 μg/g) [79]. This indicates that decoration material stores are a major source of DMP contamination in indoor environments.

The concentrations of DEP in the dust from decoration material stores were relatively low compared to findings from other studies. Specifically, DEP levels were lower than those observed in household dust in Urumqi (1.5 μg/g) [29], Guangzhou (0.989 μg/g in the bedroom, 1.1 μg/g in the apartment, 1.31 μg/g in the living room, and 1.5 μg/g in the house) [56,57] and Chongqing (6.4 μg/g in the bedroom and 7.6 μg/g in the living room) [21], as well as in various public indoor settings in China and internationally, including offices and the workplace (1.42 μg/g in Guangzhou, 1.69 and 1.52 μg/g in Hong Kong, Guangzhou and Shenzhen) [56,57], shopping centers (2.32 μg/g) [57], hospitals (1.06 μg/g) [57], electronic factories (2.17 μg/g) [57], manufacturing plants (2.49 μg/g) [57], day care facilities (4.2 ng/g, Sweden) [69], workplaces (20 ng/g, Sweden) [69], nursery and primary schools (2.89 ng/g, France) [78], hotels (4.15 ng/g, Saudi Arabia) [80], hospitals (7.0 ng/g, Qatar) [81] and cars (4.7 ng/g, Saudi Arabia) [76]. DEP is primarily used in pharmaceutical and personal-care products, which are major contributors to its presence in the environment [30,82]. The low DEP levels in this study suggested that decoration materials are not a significant contributor to DEP in indoor environments, likely due to the limited use of such products in decoration material stores [30].

The levels of DOP in this study were relatively low, consistent with other studies where concentrations were generally below 13 μg/g. Notably, in Taiwan, DOP concentrations were much higher, with readings of 81.1 μg/g at home, 180.5 μg/g in kindergartens, and 212.4 μg/g in elementary schools [59]. In contrast, the concentration of BBP in decoration material stores in our study was only detected in flooring stores (0.141 μg/g), which is similar to or lower than values reported in most studies, ranging from not-detected to 105 μg/g.

### 3.4. Source Elucidation

**Figure 2:**
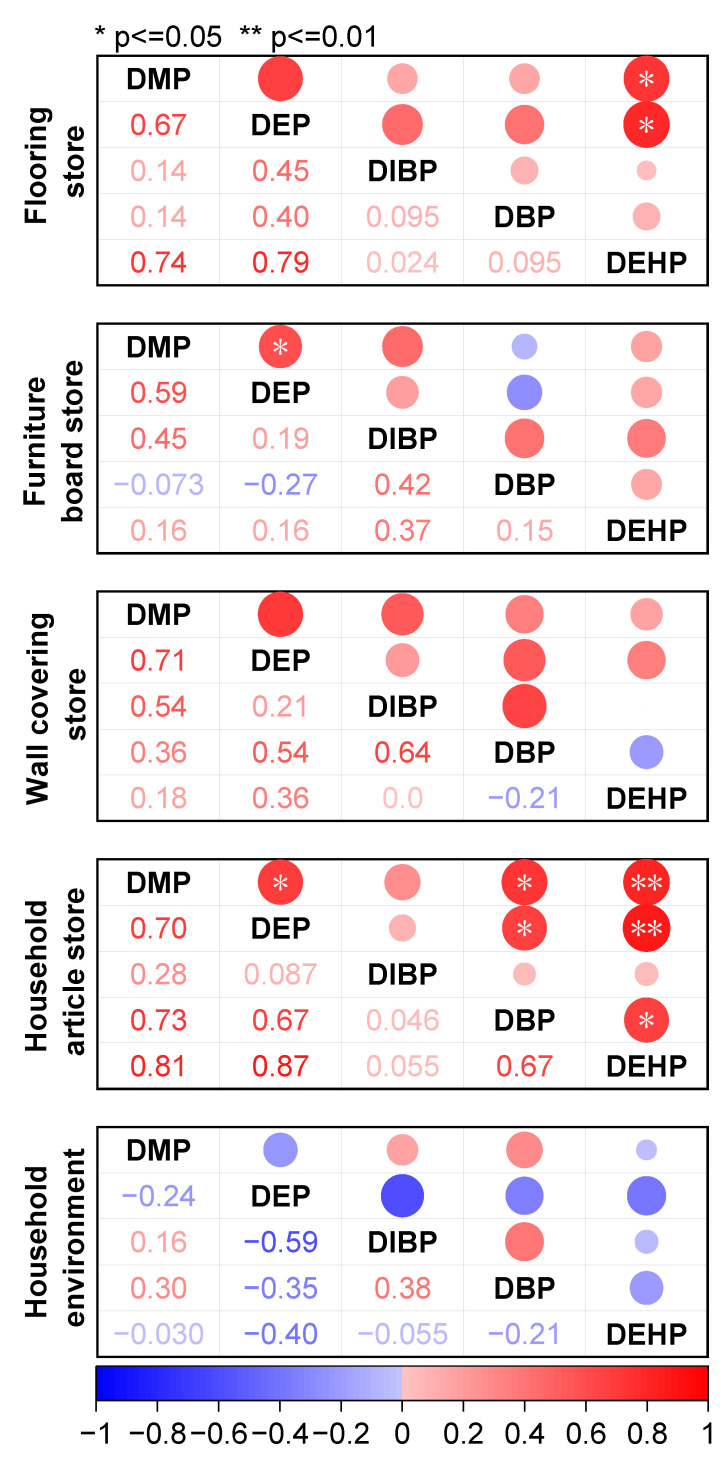
Correlation analysis of PAE concentrations in the dust from different decoration-material stores and the household environment.

To provide a clearer understanding of the sources of PAEs in different types of decoration material stores and their associations with specific PAEs, we further analyzed the data and discussed the implications for PAEs originating from decoration materials. Concentration correlations of PAEs were analyzed (Tables S1 and S2). Significant correlations were observed between DMP and DEHP in the flooring store (*p* < 0.05) and DMP and DEP in the furniture board store (*p* < 0.05), as well as DMP and DEP (*p* < 0.05), DMP and DBP (*p* < 0.05), and DMP and DEHP (*p* < 0.01) in the household article store (Figure 2). Principal component analysis (PCA), a multivariate statistical technique widely employed for simplifying and interpreting large datasets, was employed to identify the sources of PAEs in dust from decoration material stores [83]. To correct the skewness inherent in environmental contaminants, PAE concentrations were log-transformed before performing PCA. Loadings (coefficients) exceeding 60% of the maximum coefficient in absolute value for each principal component were considered significant. The resulting PCA loading values and sample scores were utilized to illustrate relationships among variables. For each decoration material store, two principal components were extracted, as detailed in Table S3.

**Figure 3:**
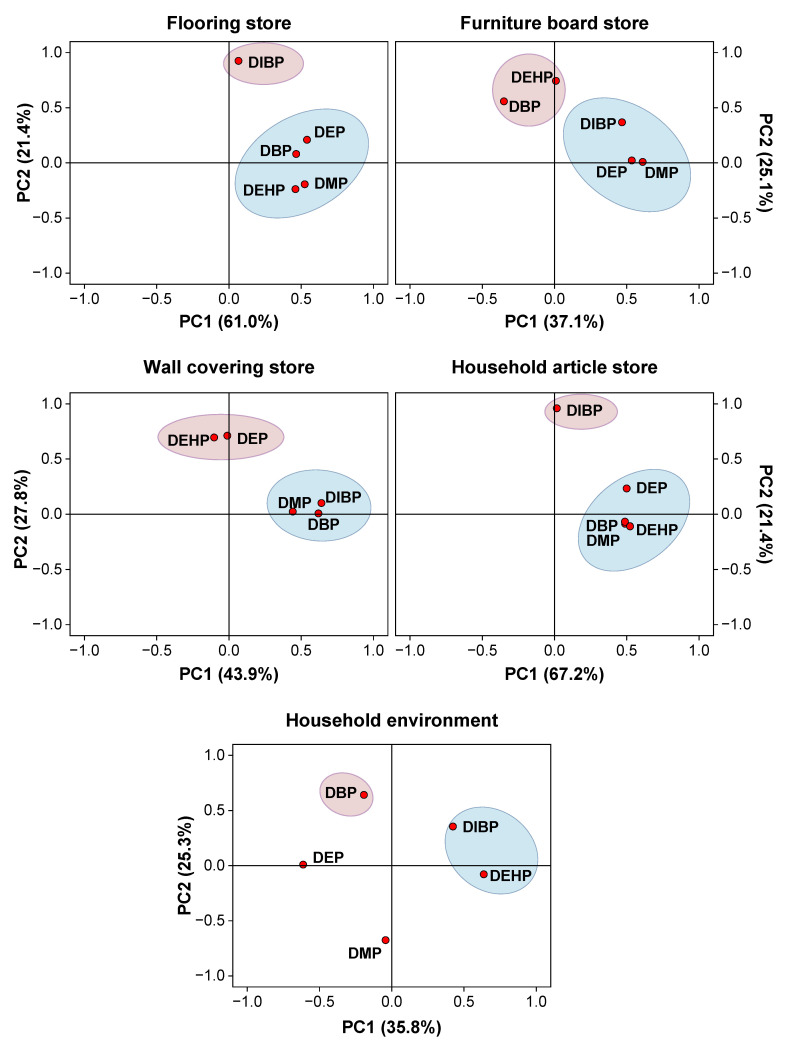
Principle component analysis of PAE concentrations in the dust from different decoration-material stores and the household environment.

In flooring stores, PC 1, accounting for 61.0% of the total variability, was mainly influenced by DMP, DEP, DBP, and DEHP, while PC 2, accounting for 21.4% of the total variability, was predominantly associated with DIBP (Figure 3). The flooring available in flooring stores typically included solid wood flooring, laminated flooring, and particleboard flooring. As mentioned earlier, the levels of DMP in dust from decoration material stores can serve as an indicator of PAE pollution originating from decoration materials. Therefore, it can be inferred that PAEs aggregated into the same principal components as DMP may originate from identical or similar sources. Among consumers, DMP and DEP are commonly used as solvents, while DBP and DEHP serve as plasticizers [84]. Previous studies have shown that DMP is the predominant PAE in various paints and waterborne coatings [85]. Additionally, workers in PVC flooring factories have been found to be exposed to high concentrations of DEHP and DBP, and painters have been exposed to DBP at significantly higher levels compared to the general population [47,86,87]. During the manufacturing process, water-based paint coatings, finishes and varnishes, which mainly contain DMP, DEP, DBP, and DEHP are often applied to flooring surfaces to enhance their appearance [85,88]. Consequently, DMP, DEP, DBP, and DEHP in dust of flooring stores may be attributed to the application of these paint coatings, finishes and varnishes. Moreover, in the production process, the surface of the floor usually needs to be coated with a layer of plastic film to increase its surface hardness, water resistance and wear resistance. DBP and DEHP are commonly used as plasticizers in plastics, especially in PVC materials [84]. Therefore, DBP, and DEHP in the dust of flooring stores may also originate from the plastic materials on the floor surface. As a result, PC 1 may represent a composite source including paint coatings, finishes, varnishes and plastic materials. PC 2 had a strong weighting for DIBP, likely due to its release from compressed-wood flooring. Compressed wood, known for its excellent mechanical properties, is often used as an adhesive in wood products and joints [89]. High DBP content in household dust has been linked to compressed-wood flooring. A Japanese study found the highest concentrations of DIBP in dust from compressed-wood floor surfaces, indicating that compressed wood may be the primary source of DIBP [90]. This aligns with the strong weighting of DIBP in PC 2, likely due to its release from compressed-wood flooring [90].

In furniture board stores, PC 1, which accounted for 37.1% of the total variance, was heavily loaded with DMP, DEP, and DIBP. PC 2, accounting for 25.1% of the total variance, was heavily loaded with DEHP and DBP. Similar to the flooring store, PC 1 may represent paint coatings, finishes and varnishes used in the manufacture and surface treatment of furniture panels, and PC 2 represents plastic materials on the surface of furniture board. The principal component analysis of various PAEs in the dust of flooring and furniture board stores showed some differences, likely stemming from variations in their manufacturing processes, usage environments, and material compositions. In manufacturing, both flooring and furniture boards typically comprise wood, fiberboard, or other substrates along with plastic materials, albeit with potentially different proportions and types. Furniture boards may lean towards using engineered wood products like plywood or medium-density fiberboard, while flooring might favor wood–plastic composites or materials coated with plastic films, each potentially containing varying types and concentrations of PAEs, thus leading to differences in PAE sources. Furthermore, due to differing usage environments, flooring may require materials more resistant to wear, water, and corrosion, while furniture boards may prioritize aesthetics and decorative effects. Consequently, the types and concentrations of PAEs added during material manufacturing, as well as their release and migration during use, may vary accordingly.

In wall covering stores, PC 1, explaining 43.9% of total variance, showed high loadings for DMP, DIBP and DBP. PAEs are the primary additives used in the textile industry. They are mainly used in textile-coating finishing, softening finishing, plasticizing sol printing, and material dyeing, to improve the softness, plasticity and adhesion of materials, coating, and printing. Liang et al. investigated the content of PAEs across different units of four textile-dyeing wastewater plants in China, revealing high concentrations of DMP and DBP [91]. Previous studies have identified DMP and DBP as the most concentrated PAEs found in textile-dyeing wastewater [91]. Additionally, DIBP has been frequently detected in textiles from southern-European and northern-African countries [92]. These findings indicated that emissions during the production of printing and dyeing coatings for wall coverings are likely primary sources of DMP, DIBP and DBP. PC 2, explaining 27.8% of total variance, was heavily loaded with DEHP and DEP. The origins of DEHP and DEP in wall covering stores remain somewhat unclear. PVC materials, which are frequently used in the production of wall coverings, often exhibit high concentrations of DEHP, suggesting that these PVC materials used in wall coverings could serve as significant DEHP sources [93].

In household article stores, PC 1 explained 67.2% of the total variance and was mainly influenced by DMP, DEP, DBP and DEHP. PC 2, which accounted for 21.4% of the total variance, was predominantly weighted by DIBP. The results for PCA in household article stores showed similarities with those in flooring stores. However, the household goods stores presented a wide array of intricate products, including flooring, furniture board, wallcovering and various home accessories, making it challenging to identify the sources of PAEs in these establishments. Based on the analyzed result, we hypothesized that DMP, DEP, DBP and DEHP, which were associated with PC 1, may originate from the release of these compounds from decoration materials, while DIBP, linked to PC 2, likely comes from other sources.

Compared to decoration material stores, the sources of PAEs in household settings dust are more complex. In indoor environments, PAE pollution primarily originates from the release of PAEs in everyday consumer goods. Consequently, lifestyle and the use of consumer products play a significant role in determining the sources of PAEs in the environment. The results for PCA revealed distinct aggregation trends of PAEs in household dust, and no significant correlation among the concentrations of different PAEs was observed. In the household environment, PC 1, capturing 35.8% of the total variance, was predominantly influenced by DIBP and DEHP. These compounds are commonly utilized in plastic consumer goods, suggesting that PC 1 is primarily associated with the leaching of PAEs from plastic products [90,94]. PC 2, accounting for 25.3% of the total variance, was predominantly characterized by DBP, which is commonly found in commercial fragrances and latex paint which is extensively used in everyday life [95,96]. Therefore, commercial products are considered the primary source of DBP in household dust. Conversely, DMP and DEP were not significant contributors to PC 1 and PC 2. However, they are likely derived from personal care products and other consumer goods [30]. The principal-component aggregation trends of PAEs in household dust differ notably from those in decoration material stores, and there is no significant correlation among the concentrations of different PAEs. This diversity is primarily due to the varied indoor sources of PAEs, which include not only decoration materials but also a wide range of consumer products such as plastics and personal care items.

### 3.5. Exposure Risk Assessment

#### 3.5.1. Estimation of Exposure Doses

**Figure 4:**
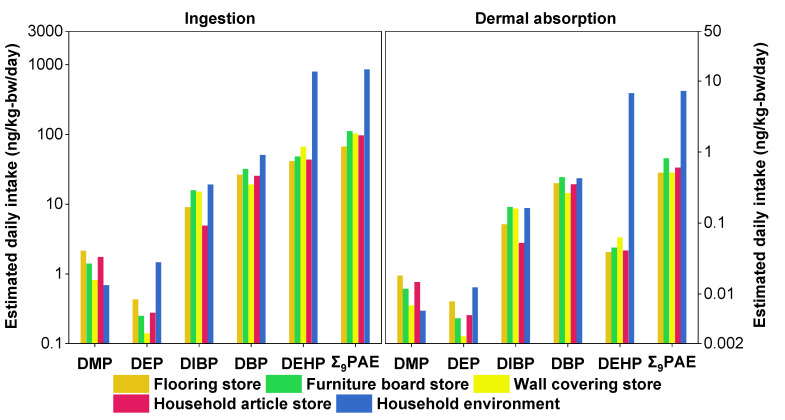
Estimated daily intakes of PAEs via non-dietary dust ingestion and dermal-absorption pathways.

To estimate the daily intakes of PAEs from dust in different decoration-material stores, the EDIs of PAEs via ingestion and dermal-absorption pathways were calculated (Figure 4 and Table S4). Ingestion is identified as the primary pathway of human exposure to PAEs through dust, as the total EDIs of nine PAEs, as well as the EDI of each individual PAE via ingestion, were significantly higher than those via dermal absorption for all groups studied. The EDIs of PAEs via ingestion and dermal-absorption pathways for flooring store staff (66.6 ng/kg-bw/day and 0.508 ng/kg-bw/day) were lower than those for staff in wall covering stores (102 ng/kg-bw/day and 0.509 ng/kg-bw/day), furniture board stores (111 ng/kg-bw/day and 0.813 ng/kg-bw/day) and household articles (96.5 ng/kg-bw/day and 0.601 ng/kg-bw/day). Notably, all these values were significantly lower than those observed for residents living in a household environment (850 ng/kg-bw/day and 7.19 ng/kg-bw/day). This indicated that general indoor environments have additional sources of PAEs and pose a higher risk of PAE exposure than specific decoration-material stores.

The primary PAE of concern is DEHP, which showed varying levels of EDIs across different store types. The total EDIs of DEHP varied among different types of stores, with wall covering stores having the highest EDIs (66.3 ng/kg-bw/day) and flooring stores having the lowest (41.2 ng/kg-bw/day). This difference in DEHP levels may stem from variations in workplace exposure and differences in work practices and materials used in these stores. DBP and DIBP were also major PAEs contributing to daily ingestion intake. For ingestion, the EDIs of DBP were highest among furniture board-store staff (31.9 ng/kg-bw/day) and lowest among wall covering-store staff (19.1 ng/kg-bw/day). In contrast, the ingestion EDIs of DIBP were highest for furniture board-store staff (15.7 ng/kg-bw/day) and lowest for household article-store staff (4.91 ng/kg-bw/day). Dermal-absorption EDIs also showed variability, with DBP levels peaking among flooring store staff (0.363 ng/kg-bw/day) and being lowest among furniture board-store staff (0.167 ng/kg-bw/day). For DIBP, dermal absorption was highest in furniture board-store staff (0.439 ng/kg-bw/day) and lowest in household article-store staff (0.0523 ng/kg-bw/day). When comparing these values to PAE exposure through foodstuffs, it is evident that exposure from dust in decoration material stores, while lower, still constitutes a significant portion of overall exposure. For instance, the ingestion EDIs of DBP and DIBP in these environments accounted for 8–13% and 2–6%, respectively, of the corresponding EDIs from food sources (243 ng/kg-bw/day for DBP and 245 ng/kg-bw/day for DIBP) [97]. Moreover, the EDIs of DEHP from dermal absorption are notably lower across all store types, indicating that DBP and DIBP are the more significant contributors to PAE exposure in these occupational settings. The median EDIs of DMP, DEP, DHxP, BBP, DCHP and DOP were relatively low. The EDIs of other PAEs were approximately 1–2 orders-of-magnitude lower than those from foodstuffs for adults (16.5 ng/kg-bw/day for DMP, 13.6 ng/kg-bw/day for DEP, and 10.0 ng/kg-bw/day for BBP) [97].

#### 3.5.2. Cumulative Risk Assessment of Exposure to PAEs

Cumulative risk assessment was applied to estimate the exposure risk to DIBP, DBP, and DEHP based on the reference dose (RfD) recommended by the United States Environmental Protection Agency (U.S. EPA) and the reference dose for anti-androgenicity (RfD AA) developed by Kortenkamp and Faust [36]. An HI value lower than 1 is considered safe [37]. Overall, the HI values based on RfD were higher than those based on RfD AA, indicating that using the RfD AA, which specifically addresses anti-androgenic effects, results in a lower estimation of risk compared to the general RfD (Table S4). The relative-exposure levels to DIBP, DBP, and DEHP vary significantly among different types of decoration material stores. The HI values of DIBP, DBP, and DEHP for staff in flooring stores, wall covering stores, furniture board stores, and household article stores ranged from 2.54 × 10^−4^ to 4.15 × 10^−3^ (median 1.57 × 10^−3^), 1.22 × 10^−3^ to 6.18 × 10^−3^ (median 2.47 × 10^−3^), 3.19 × 10^−4^ to 3.82 × 10^−3^ (median 2.15 × 10^−3^), and 7.32 × 10^−4^ to 6.19 × 10^−3^ (median 2.18 × 10^−3^), respectively. Despite variations in HI values, all HI values were below 1, implying that, according to the criteria set, the exposure levels for all decoration material-store staff are within the acceptable safety limits for both RfD and RfD AA. It is worth noting that across all types of stores, wall covering-store staff had the highest median HI values (0.0035 based on RfD and 0.0025 based on RfD AA), while flooring store staff had the lowest (0.0022 and 0.0016), regardless of whether RfD or RfD AA was used. This indicates that wall covering-store staff are exposed to higher levels of DIBP, DBP, and DEHP, which may pose a greater potential risk to their health compared to flooring store staff.

#### 3.5.3. Carcinogenic Risk Assessment and China-Specific NSRL and MADL Risk 

##### Assessments of DEHP

The median CR values of DEHP for staffs from wall covering stores, flooring stores, furniture board stores, and household article stores were 5.57 × 10^−7^, 346 × 10^−7^, 4.02 × 10^−7^, and 3.65 × 10^−7^, respectively. Compared to the threshold value of CR (1 × 10^−5^), none of the values exceeded the threshold, indicating that staff in all decoration-material stores are not at risk of carcinogenic effects from exposure [98,99]. Given the carcinogenic potential and high exposure levels of DEHP, risk quotients (RQs) were calculated in the risk assessment of DEHP, using reference values from China-specific No Significant Risk Level (NSRL) and Maximum Allowable Dose Level (MADL). The NSRL for DEHP was estimated for both male and female decoration-material-store staff through dust ingestion and dermal-absorption pathways, while MADL for DEHP was only estimated for males through dust ingestion [22] (Table S4). The median RQ values for staff in flooring stores, furniture board stores, and household article stores were 0.00931, 0.0108, and 0.00982, respectively, which were approximately 1.5 times lower than those for wall covering-store staff (0.0150). Similarly, the median DEHP RQ values based on MADL for male stuff in flooring stores, furniture board stores, and household article stores were about 1.5 times lower than that for wall covering-store staff. The median RQ values for DEHP, based on NSRL, showed a consistent pattern. However, while all CR and RQ values are below the threshold, wall covering-store staff exhibit the highest median CR value (5.57 × 10^−7^) and the highest RQ values (0.0150 and 0.0114). This indicated that this group is subject to a relatively higher risk from DEHP compared to other store staff. Therefore, there is a need for targeted risk-management strategies to further reduce DEHP exposure in wall covering.

## 4. Conclusions

This study provides insights into the levels, sources, exposure doses, and potential health risks associated with PAE contamination in dust from decoration material stores. By collecting and analyzing dust samples from different types of decoration material stores (flooring, furniture boards, wall coverings, and household articles), our study quantifies the concentrations of PAEs, identifies the predominant types, understands their sources within indoor environments, estimates exposure doses for staff through non-dietary ingestion and dermal-absorption pathways, and assesses the associated health risks. It underscores the importance of considering dust exposure in decoration material stores as a noteworthy route of PAE exposure for humans. Although the exposure levels are lower than those from dietary sources, they are still substantial enough to warrant attention, especially given the potential health risks associated with prolonged and cumulative exposure to PAEs. This highlights the need for targeted interventions and protective measures to reduce occupational exposure to these harmful chemicals. There are several limitations that warrant consideration. Firstly, despite collecting 50 samples, the study scope was limited, and future research should increase the sample size to improve the representativeness of results. Secondly, samples were collected from a specific region only, and the results may not be applicable to other geographical areas. Future research should consider a broader geographical range to enhance the generalizability of findings. Additionally, this study only evaluated PAE exposure in dust, while other potential exposure pathways such as air sources of PAEs were not included in the analysis. Lastly, the study primarily assessed short-term exposure, and the health impacts of long-term exposure remain insufficiently researched.

## Figures and Tables

**Table 1:** Concentrations and detection frequencies of PAEs in dust from decoration material stores (ng/g).

Store	Value	DMP	DEP	DIBP	DBP	DHxP	BBP	DEHP	DCHP	DOP	Total
Flooring (n = 8)	Min	530	92.9	2710	12,700	nd *^a^*	nd	nd	nd	nd	49,600
	Median	3060	616	12,900	37,700	nd	141	56,000	17.3	nd	95,300
	Max	7560	2640	21,600	248,000	4.72	522	114,000	161	916	374,000
	DR *^b^*	100%	100%	100%	100%	38%	63%	88%	63%	25%	
Furniture board (n = 14)	Min	87.6	24.4	1800	29,500	nd	nd	nd	nd	nd	65,800
	Median	2000	356	22,500	45,600	nd	nd	68,500	10.7	nd	159,000
	Max	9990	1990	319,000	132,000	21.9	136	127,000	268	1320	460,000
	DR	100%	100%	100%	100%	29%	29%	93%	50%	7%	
Wall covering (n = 7)	Min	337	117	5540	9690	nd	nd	36,000	nd	nd	86,600
	Median	1160	199	21,500	27,400	nd	nd	94,900	13.4	nd	146,000
	Max	5340	1380	54,400	542,000	2.14	110	238,000	181	nd	695,000
	DR	100%	100%	100%	100%	29%	29%	100%	57%	0%	
Household article (n = 11)	Min	621	72.4	nd	11,900	nd	nd	26,200	nd	nd	46,100
	Median	2480	396	7030	36,400	2.33	nd	62,200	nd	nd	138,000
	Max	5850	983	202,000	245,000	38.5	nd	193,000	180	nd	445,000
	DR	100%	100%	82%	100%	73%	0%	100%	45%	0%	
Household environment (n = 10)	Min	nd	676	19,500	40,700	nd	nd	nd	nd	nd	427,000
	Median	979	2090	27,300	72,100	nd	nd	1,130,000	nd	nd	1,220,000
	Max	9190	4880	209,000	585,000	nd	33,800	7,630,000	nd	nd	7,820,000
	DR	90%	100%	100%	100%	0%	20%	80%	0%	0%	

Note: *^a^* not detected; *^b^* detection rate.

## Data Availability

Data are contained within the article.

## Supplementary Materials

This material is available free of charge via the Internet at https://www.mdpi.com/article/10.3390/toxics12070505/s1, Table S1: Concentration correlations of PAEs in dust from decoration material stores; Table S2: Correlation of PAE concentrations in indoor dust between different decoration-material stores; Table S3: Principle-component analysis of PAE concentrations in indoor dust from different decoration-material stores; Table S4: Estimated daily intakes of PAEs via non-dietary dust ingestion and dermal-absorption pathways (ng/kg-bw/day); Table S5: Carcinogenic risk assessment and China-specific NSRL and MADL risk assessments of DEHP; Figure S1: Concentrations of PAEs in the dust from different decoration-material stores.
