# Supplementary material for: Phthalate Acid Esters (PAEs) in Indoor Dust from Decoration Material Stores: Occurrence, Sources, and Health Risks
Source: Toxics. 2024 Jul 13;12(7):505. doi: 10.3390/toxics12070505 (PMC11280923; doi:10.3390/toxics12070505)
Supplement: Supplementary file 1 [file toxics-12-00505-s001.zip › toxics-3091913-supplementary.pdf]

Supplementary Materials

Phthalate Acid Esters (PAEs) in Indoor Dust from Decoration Material Stores: Occurrence, Sources, and Health Risks

Li-Bo Chen [1], Chong-Jing Gao [1,2,*], Ying Zhang [1], Hao-Yang Shen [1], Xin-Yu Lu [1], Cenyan Huang [1], Xiaorong Dai [1], Jien Ye [1], Xiaoyu Jia [3], Kun Wu [3], Guojing Yang [1], Hang Xiao [4, 5], Wan-Li Ma [6,7,*]

[1] College of Biological & Environmental Science, Zhejiang Wanli University, Ningbo, 315100, China

[2] State Key Laboratory of Urban Water Resource and Environment, Harbin Institute of Technology, Harbin 150090, China.

[3] Ningbo Observation and Research Station, Institute of Urban Environment, Chinese Academy of Sciences, Ningbo 315830, China

[4] Center for Excellence in Regional Atmospheric Environment, Institute of Urban Environment, Chinese Academy of Sciences, Xiamen 361021, China

[5] Ningbo (Beilun) Zhongke Haixi Industrial Technology Innovation Center, Ningbo 315021, P. R. China

[6] International Joint Research Center for Persistent Toxic Substances (IJRC-PTS), State Key Laboratory of Urban Water Resource and Environment, Harbin Institute of Technology, Harbin 150090, China

[7] Heilongjiang Provincial Key Laboratory of Polar Environment and Ecosystem (HPKL-PEE), Harbin 150090, China

Table S1. Concentration correlations of PAEs in dust from decoration material stores.

Types	Compound	DMP	DEP	DIBP	DBP	DEHP
Flooring	DMP	1.000	0.667	0.143	0.143	0.738*
	DEP		1.000	0.452	0.405	0.786*
	DIBP			1.000	0.095	0.024
	DBP				1.000	0.095
	DEHP					1.000
Furniture board	DMP	1.000	0.587*	0.451	-0.073	0.160
	DEP		1.000	0.187	-0.266	0.156
	DIBP			1.000	0.415	0.371
	DBP				1.000	0.152
	DEHP					1.000
Wall covering	DMP	1.000	0.714	0.536	0.357	0.179
	DEP		1.000	0.214	0.536	0.357
	DIBP			1.000	0.643	0.000
	DBP				1.000	-0.214
	DEHP					1.000
Household article	DMP	1.000	0.700*	0.278	0.727*	0.809**
	DEP		1.000	0.087	0.673*	0.873**
	DIBP			1.000	0.046	0.055
	DBP				1.000	0.673*
	DEHP					1.000
Household environment	DMP	1.000	-0.236	0.164	0.297	-0.030
	DEP		1.000	-0.588	-0.345	-0.395
	DIBP			1.000	0.382	-0.055
	DBP				1.000	-0.213
	DEHP					1.000

Note: * means $p < 0.05$, ** means $p < 0.01$.

Table S2. Correlation of PAE concentrations in indoor dust between different decoration materials stores.

Compound	Types	Flooring	Furniture board	Wall covering	Household article	Household environment
DMP	Flooring	1.000	0.024	-0.143	-0.381	-0.119
	Furniture board		1.000	0.500	0.300	-0.006
	Wall covering			1.000	0.643	-0.429
	Household article				1.000	-0.503
	Household environment					1.000
DEP	Flooring	1.000	0.071	-0.571	0.357	0.357
	Furniture board		1.000	-0.107	-0.100	0.261
	Wall covering			1.000	0.000	0.036
	Household article				1.000	0.248
	Household environment					1.000
DIBP	Flooring	1.000	0.000	0.036	-0.214	-0.024
	Furniture board		1.000	-0.357	-0.237	0.321
	Wall covering			1.000	0.179	-0.107
	Household article				1.000	-0.310
	Household environment					1.000
DBP	Flooring	1.000	-0.905**	0.393	0.452	0.381
	Furniture board		1.000	-0.321	-0.409	-0.079
	Wall covering			1.000	0.071	0.571
	Household article				1.000	0.006
	Household environment					1.000
DEHP	Flooring	1.000	0.119	-0.429	0.238	-0.714*
	Furniture board		1.000	0.036	0.118	0.055
	Wall covering			1.000	-0.500	0.607
	Household article				1.000	0.225
	Household environment					1.000

Note: * means $p < 0.05$, ** means $p < 0.01$.

Table S3. Principle component analysis of PAEs concentrations in indoor dust from different decoration material stores.

Compound	Flooring		Furniture board		Wall covering		Household article		Household environment	
	PC1	PC2	PC1	PC2	PC1	PC2	PC1	PC2	PC1	PC2
DMP	0.916	-	0.831	0.009	0.655	0.028	0.895	-	-	-
DEP	0.943	0.217	0.728	0.024	-	0.838	0.916	0.241	-	0.010
DIBP	0.115	0.957	0.636	0.412	0.949	0.119	0.029	0.994	0.566	0.398
DBP	0.813	0.083	-	0.625	0.918	0.008	0.894	-	-	0.721
DEHP	0.804	-	0.013	0.833	-	0.819	0.959	-	0.852	-
Cumulativ	61.0	21.4	37.1	25.1	43.9	27.8	67.2	21.4	35.8	25.3

Table S4. Estimated daily intakes of PAEs via non-dietary dust ingestion and dermal absorption pathways (ng/kg-bw/day).

Store		DMP	DEP	DIBP	DBP	DHxP	BBP	DEHP	DCHP	DOP	Σ_9PAEs
Ingestion											
Flooring store	Min	0.370	0.0649	1.89	8.84	<0.001	<0.001	<0.001	<0.001	<0.001	34.6
	Median	2.13	0.430	9.00	26.4	<0.001	0.0985	41.2	0.0121	<0.001	66.6
	Max	5.28	1.85	15.1	173	0.00330	0.365	79.6	0.113	0.640	261
Furniture board store	Min	0.0612	0.0171	1.26	20.6	<0.001	<0.001	<0.001	<0.001	<0.001	46.0
	Median	1.40	0.249	15.7	31.8	<0.001	<0.001	47.9	0.00748	<0.001	111
	Max	6.98	1.39	223	92.1	0.0153	0.0947	88.4	0.188	0.919	321
Wall covering store	Min	0.235	0.0819	3.87	6.77	<0.001	<0.001	25.2	<0.001	<0.001	60.5
	Median	0.813	0.139	15.0	19.1	<0.001	<0.001	66.3	0.00936	<0.001	102
	Max	3.73	0.966	38.0	379	0.00149	0.0767	166	0.126	<0.001	485
Household article store	Min	0.433	0.0505	<0.001	8.31	<0.001	<0.001	18.3	<0.001	<0.001	32.2
	Median	1.73	0.277	4.91	25.4	0.00163	<0.001	43.4	<0.001	<0.001	96.5
	Max	4.09	0.687	141	171	0.0269	<0.001	134	0.126	<0.001	310
Household environment	Min	<0.001	0.472	13.6	28.4	<0.001	<0.001	<0.001	<0.001	<0.001	298
	Median	0.684	1.46	19.1	50.4	<0.001	<0.001	791	<0.001	<0.001	850
	Max	6.42	3.41	146	409	<0.001	23.6	5330	<0.001	<0.001	5460
Dermal											
Flooring store	Min	0.00313	0.00118	0.0202	0.122	<0.001	<0.001	<0.001	<0.001	<0.001	0.244
	Median	0.0181	0.00782	0.0958	0.363	<0.001	<0.001	0.0387	<0.001	<0.001	0.508
	Max	0.0447	0.0335	0.161	2.39	<0.001	0.00228	0.0747	<0.001	<0.001	2.63
	Min	0.000518	0.000310	0.0134	0.285	<0.001	<0.001	<0.001	<0.001	<0.001	0.346

Furniture board store	Median	0.0118	0.00452	0.167	0.439	<0.001	<0.001	0.045	<0.001	<0.001	0.813
	Max	0.0590	0.0253	2.37	1.27	<0.001	<0.001	0.083	<0.001	<0.001	2.86
Wall covering store	Min	0.00199	0.00149	0.0412	0.0933	<0.001	<0.001	0.024	<0.001	<0.001	0.217
	Median	0.00688	0.00253	0.160	0.264	<0.001	<0.001	0.062	<0.001	<0.001	0.509
	Max	0.0316	0.0176	0.405	5.22	<0.001	<0.001	0.156	<0.001	<0.001	5.71
Household article store	Min	0.00367	0.000919	<0.001	0.115	<0.001	<0.001	0.017	<0.001	<0.001	0.161
	Median	0.0147	0.00503	0.0523	0.350	<0.001	<0.001	0.041	<0.001	<0.001	0.601
	Max	0.0346	0.0125	1.51	2.36	<0.001	<0.001	0.126	<0.001	<0.001	2.53
Household environment	Min	<0.001	0.00400	0.115	0.241	<0.001	<0.001	<0.001	<0.001	<0.001	2.52
	Median	0.00578	0.0123	0.161	0.426	<0.001	<0.001	6.692	<0.001	<0.001	7.19
	Max	0.0543	0.0288	1.24	3.46	<0.001	0.199	45.077	<0.001	<0.001	46.2
Total											
Flooring store	Min	0.373	0.0661	1.91	8.96	<0.001	<0.001	<0.001	<0.001	<0.001	35.0
	Median	2.15	0.438	9.09	26.7	<0.001	0.0991	41.2	0.0121	<0.001	66.9
	Max	5.32	1.88	15.3	176	0.00330	0.367	79.6	0.113	0.641	264
Furniture board store	Min	0.0617	0.0174	1.27	20.9	<0.001	<0.001	<0.001	<0.001	<0.001	46.5
	Median	1.41	0.253	15.9	32.3	<0.001	<0.001	47.9	0.00749	<0.001	112
	Max	7.03	1.42	225	93.4	0.0153	0.0953	88.5	0.188	0.920	324
Wall covering store	Min	0.237	0.0833	3.91	6.86	<0.001	<0.001	25.2	<0.001	<0.001	60.7
	Median	0.819	0.142	15.2	19.4	<0.001	<0.001	66.3	0.00937	<0.001	103
	Max	3.76	0.984	38.4	384	0.00149	0.0772	167	0.127	<0.001	491
Household article store	Min	0.437	0.0515	<0.001	8.42	<0.001	<0.001	18.3	<0.001	<0.001	32.3
	Median	1.75	0.282	4.96	25.7	0.00163	<0.001	43.5	<0.001	<0.001	96.9
	Max	4.12	0.699	143	173	0.0269	<0.001	135	0.126	<0.001	313

Household environment	Min	<0.001	0.476	13.8	28.7	<0.001	<0.001	<0.001	<0.001	<0.001	301
	Median	0.689	1.47	19.2	50.8	<0.001	<0.001	798	<0.001	<0.001	857
	Max	6.47	3.44	147	412	<0.001	23.8	5370	<0.001	<0.001	5500

Table S5. Carcinogenic risk assessment and China specific NSRL and MADL risk assessments of DEHP.

Store		HI (RfDs)	HI (RfDs AA)	CR	NSRL	MADL
Flooring store	Min	1.99×10^{-4}	2.54×10^{-4}	0	0	0
	Median	2.23×10^{-3}	1.57×10^{-3}	3.46×10^{-7}	0.00931	0.00710
	Max	5.30×10^{-3}	4.15×10^{-3}	6.68×10^{-7}	0.0180	0.0137
Furniture board store	Min	2.45×10^{-4}	3.19×10^{-4}	0	0	0
	Median	2.80×10^{-3}	2.15×10^{-3}	4.02×10^{-7}	0.0108	0.00825
	Max	5.21×10^{-3}	3.82×10^{-3}	7.43×10^{-7}	0.0200	0.0152
Wall covering store	Min	1.57×10^{-3}	1.22×10^{-3}	2.11×10^{-7}	0.00569	0.00434
	Median	3.52×10^{-3}	2.47×10^{-3}	5.57×10^{-7}	0.0150	0.0114
	Max	8.50×10^{-3}	6.18×10^{-3}	1.40×10^{-6}	0.0376	0.0287
Household article store	Min	1.01×10^{-3}	7.32×10^{-4}	1.54×10^{-7}	0.00413	0.00315
	Median	2.65×10^{-3}	2.18×10^{-3}	3.65×10^{-7}	0.00982	0.00749
	Max	8.43×10^{-3}	6.19×10^{-3}	1.13×10^{-6}	0.0304	0.0232
Household environment	Min	1.44×10^{-3}	1.99×10^{-3}	0	0	0
	Median	4.00×10^{-2}	2.68×10^{-2}	6.64×10^{-6}	0.180	0.136
	Max	2.67×10^{-1}	1.78×10^{-1}	4.47×10^{-5}	1.21	0.918

Note: HI values were estimated only based on PAEs concentrations in decoration materials stores and household environment dust.

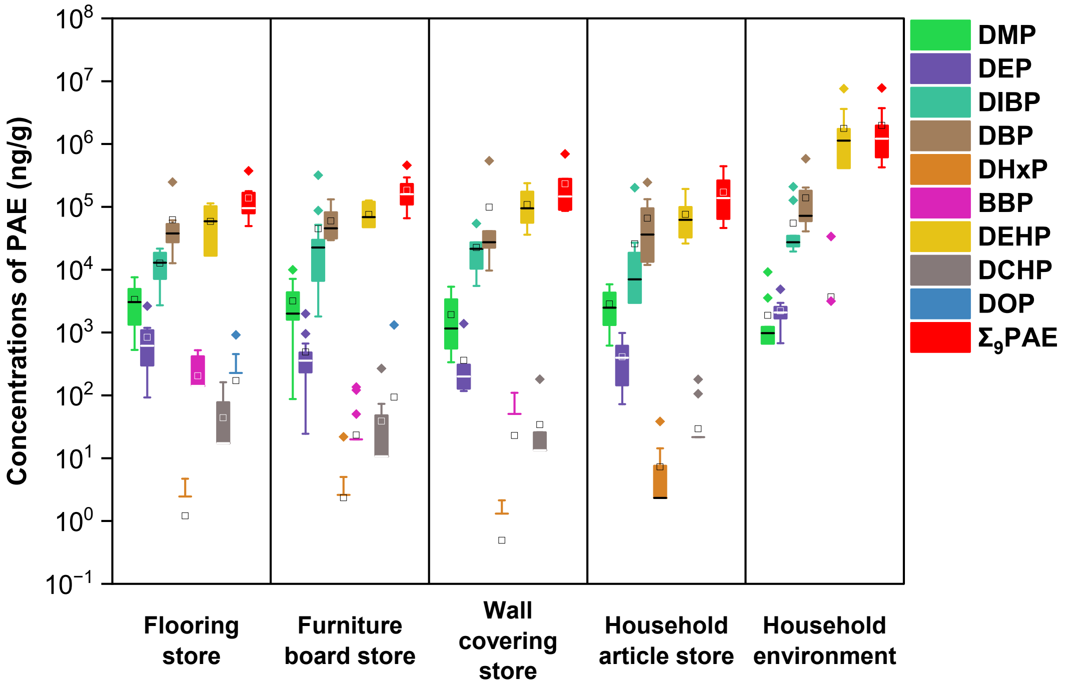

Figure S1. Concentrations of PAEs in the dust from different decoration material stores.
